# Phase 3 THOR Japanese subgroup analysis: erdafitinib in advanced or metastatic urothelial cancer and fibroblast growth factor receptor alterations

**DOI:** 10.1007/s10147-024-02583-3

**Published:** 2024-07-17

**Authors:** Nobuaki Matsubara, Yuji Miura, Hiroyuki Nishiyama, Rikiya Taoka, Takahiro Kojima, Nobuaki Shimizu, Jason Hwang, Tatsuya Ote, Ryo Oyama, Kiichiro Toyoizumi, Sutapa Mukhopadhyay, Spyros Triantos, Kris Deprince, Yohann Loriot

**Affiliations:** 1 Department of Medical Oncology, National Cancer Center Hospital East, 6-5-1 Kashiwanoha, Kashiwa, Chiba 277-8577 Japan; 2 Department of Medical Oncology, Toranomon Hospital, 2-2-2 Toranomon, Minato-ku, Tokyo, 105-8470 Japan; 3 Department of Urology, Institute of Medicine, University of Tsukuba, 1-1-1 Tennodai, Tsukuba, Ibaraki 305-8575 Japan; 4 Department of Urology, Faculty of Medicine, Kagawa University, 1750-1 Ikenobe, Miki-cho, Kita-gun, Kagawa, 761-0793 Japan; 5 Department of Urology, Aichi Cancer Center Hospital, 1-1 Kanokoden, Chikusa-ku, Nagoya, Aichi 464-8681 Japan; 6 Department of Urology, Gunma Prefectural Cancer Center, 3-39-22 Showa-machi, Maebashi, Gunma 371-8511 Japan; 7 Department of Medical Affairs, Janssen Pharmaceutical K.K, 5-2-3 Nishikanda, Chiyoda-ku, Tokyo, 101-0065 Japan; 8 Oncology Clinical Development Department, Clinical Science Division, Research and Development, Janssen Pharmaceutical K.K, 5-2-3 Nishikanda, Chiyoda-ku, Tokyo, 101-0065 Japan; 9 Research and Development, Janssen Pharmaceutical K.K, 5-2-3 Nishikanda, Chiyoda-ku, Tokyo, 101-0065 Japan; 10 Statistics and Decision Sciences, Research and Development, Janssen Pharmaceutical K. K, 5-2-3 Nishikanda, Chiyoda-ku, Tokyo, 101-0065 Japan; 11 Janssen Research and Development, 920 US Highway 202 S, Raritan, NJ 08807 USA; 12 Janssen Research and Development, 1400 McKean Road, Spring House, PA 19477 USA; 13 Janssen Research and Development, Turnhoutseweg 30, 2340 Beerse Anterwerpen, Belgium; 14 Department of Cancer Medicine, INSERM U981, Gustave Roussy, Universite Paris-Saclay, 94800 Villejuif, France

**Keywords:** Erdafitinib, Fibroblast growth factor receptor, Japanese subgroup, Oral tyrosine kinase inhibitor, Metastatic urothelial carcinoma

## Abstract

**Background:**

In the THOR trial (NCT03390504) Cohort 1, erdafitinib demonstrated significantly prolonged overall survival (OS) (median 12.1 versus 7.8 months) and reduced risk of death by 36% (hazard ratio 0.64, P = 0.005) compared with chemotherapy in metastatic urothelial carcinoma (mUC) patients with *FGFR* alterations who progressed after ≥ 1 prior treatments, including anti-PD-(L)1. There have been no reports of the Japanese subgroup results yet.

**Methods:**

THOR Cohort 1 randomized patients to erdafitinib once daily or docetaxel/vinflunine once every 3 weeks. Primary endpoint was OS. Secondary endpoints included progression-free survival (PFS) and objective response rate (ORR). No specific statistical power was set for this Japanese subgroup analysis.

**Results:**

Of 266 patients randomized, 27 (14 erdafitinib; 13 chemotherapy) were Japanese. Baseline characteristics were generally similar between treatments and to the overall population, except for more males, lower body weight, and more upper tract primary tumors among Japanese patients. Compared with chemotherapy, erdafitinib showed improved OS (median 25.4 versus 12.4 months), PFS (median 8.4 versus 2.9 months) and ORR (57.1% versus 15.4%). Any grade treatment-related adverse events (AEs) occurred in all patients from both arms but Grade 3/4 AEs and AEs leading to discontinuation were lower in the erdafitinib arm. No new safety signals were observed in the Japanese subgroup.

**Conclusion:**

In the Japanese subgroup, erdafitinib showed improved survival and response compared to chemotherapy, with no new safety concerns. These results support erdafitinib as a treatment option for Japanese mUC patients with *FGFR* alterations, and early *FGFR* testing after diagnosis of mUC should be considered.

**Supplementary Information:**

The online version contains supplementary material available at 10.1007/s10147-024-02583-3.

## Introduction

Urothelial carcinoma, also known as transitional cell carcinoma, is the predominant type of bladder, renal pelvis and ureter cancer [1]. Urothelial carcinoma is common, especially in white people, men, smokers, and the elderly [2]. According to a structured literature search from 2001 to 2017, the incidence of locally advanced or metastatic urothelial cancer (mUC) was 3.8, 3.8 and 2.8 per 100,000 in the US, Europe, and Japan, respectively [3]. In Japan, a total of 1,162 new in-hospital cases were reported in 2021 to be locally advanced or metastatic urothelial carcinoma, which represented 3.4% of all urothelial carcinoma cases [4]. The prognosis of locally advanced or mUC is still poor [5], with an analysis of in-hospital cases in Japan reporting a 5-year survival rate of approximately 17% [6].

In Japan, first-line treatment generally follows the principles of international guidelines in recommending platinum-based chemotherapy with or without avelumab maintenance depending on disease progression during chemotherapy [7]. Second-line treatment with pembrolizumab is recommended for anti-PD-(L)1 checkpoint inhibitor-naïve patients [8], whereas either single-agent chemotherapy or enfortumab vedotin [9], are recommended in patients who have previously received chemotherapy and an anti-PD-(L)1 checkpoint inhibitor. Although anti-PD-(L)1 checkpoint inhibitors are used for both first- and second-line treatment, only approximately one-third of patients respond and treatment options following anti-PD-(L)1 checkpoint inhibitor therapy are limited.

Fibroblast growth factor receptors (FGFR) regulate several cellular processes such as migration, proliferation, differentiation, and survival especially during embryonic development, as well as inflammation and wound healing in adults [10], *FGFR* gene alterations, especially *FGFR*2/3 mutations and fusions, are noted in many malignancies, including urothelial carcinoma, and may function as oncogenic drivers to promote carcinogenesis [11–13]. Approximately 20% of patients with advanced urothelial carcinoma have *FGFR* alterations [11], which are almost twice as frequent in patients with upper tract urothelial carcinoma [14]. Therefore, addressing *FGFR* alterations in advanced urothelial carcinoma as a treatment target is a suitable strategy to improve survival outcomes in these patients.

Erdafitinib, an oral, selective small-molecule pan-*FGFR* tyrosine kinase inhibitor, has been shown to inhibit downstream *FGFR* signal transduction and signaling, and possess potent antiproliferative activity on *FGFR*-altered cancer cell lines [10]. In an open-label, phase 2 study (BLC2001), erdafitinib was associated with an objective tumor response in 40% of previously treated patients who had locally advanced and unresectable or mUC with selected *FGFR3/2* alterations [15]. Accordingly, erdafitinib was granted accelerated approval by the US Food and Drug Administration [16]. Erdafitinib has also been approved in 17 other countries for treating adults with locally advanced or mUC with susceptible FGFR3/2 alterations but has not yet received approval in Japan.

Based on this background, the THOR study was conducted as a confirmatory study of the Phase 2 BLC2001 study and led to full US FDA approval. There have been no reports of the THOR Cohort 1 Japanese subgroup at present, so this subgroup analysis aims at clarifying the efficacy and safety results of Japanese patients enrolled in the study [17].

## Methods

### Study design and participants

THOR was an open-label, randomized, phase 3 study of erdafitinib versus chemotherapy conducted in 121 clinical sites globally, including 26 sites in Japan (ClinicalTrials.gov, NCT03390504) [17]. The study assigned patients into two cohorts based on prior treatment with an anti-PD-(L)1 checkpoint inhibitor, which compared erdafitinib with either choice of chemotherapy (docetaxel or vinflunine; Cohort 1) or with pembrolizumab (Cohort 2).

Inclusion and exclusion criteria have been published previously for the overall population. Patients with metastatic or surgically unresectable urothelial cancer with confirmed disease progression after one or two previous treatments that included an anti-PD-(L)1 checkpoint inhibitor were also required to meet molecular eligibility criteria. Molecular eligibility was confirmed using either central or local historical FGFR test results, with tumors having at least 1 of the following alterations: FGFR2-BICC1, FGFR2-CASP7, FGFR3-TACC3, FGFR3-BAIAP2L1; or 1 of the following FGFR3 gene mutations: R248C, S249C, G370C, Y373C. Eligible patients also had ECOG performance status of 0–2.

The final study protocol and related documents were approved by the institutional review board or independent ethics committee at each centre, and the trial was done in compliance with Good Clinical Practice, the International Conference on Harmonisation, the Declaration of Helsinki, and any local regulatory requirements. All enrolled patients provided written, informed consent.

### Randomization and treatment

Patients in Cohort 1 were randomized 1:1 to erdafitinib or chemotherapy. Erdafitinib 8 mg once daily was administered orally for 21 days in a 21-day cycle with an option to up-titrate to 9 mg daily based on phosphate level measured at Cycle 1 Day 14 and in the absence of significant toxicity. Patients assigned to chemotherapy received either vinflunine or docetaxel determined by the investigator at each site. Randomization was stratified according to the ECOG performance-status score (0 or 1 versus 2), disease distribution (presence versus absence of visceral [lung, liver, or bone] metastases), and geographic region (North America versus Europe versus the rest of the world).

Treatment with erdafitinib or chemotherapy was continued until disease progression, intolerable toxicity, withdrawal of consent or decision by the investigator to discontinue treatment, whichever occurs first. Erdafitinib dose interruptions and reductions were permitted for the management of adverse events (AEs). Guidelines for the management of specific erdafitinib toxicities such as elevated phosphate levels, dry mouth and mucositis, dry skin and skin toxicity, nail toxicity (onycholysis, onychodystrophy, paronychia), eye toxicity associated with visual changes, and dry eye can be found in the protocol of previous publication [17].

#### Endpoints and evaluation

The intention-to-treat (ITT) population, consisting of all randomised patients, was used for analysis of efficacy endpoints. The safety population, defined as patients who received at least one dose of study drug, was used for analysis of safety endpoints.

The primary endpoint was OS, defined as the time from randomization to death from any cause. The secondary endpoints of interest for this subgroup analysis were PFS (duration from randomization to disease progression or death), ORR (proportion of patients who achieved complete response or partial response), and safety. Tumor response was assessed according to RECIST version 1.1 by investigator. Adverse events were graded according to the NCI-CTCAE version 4.03.

#### Analysis method and statistical methods

Details of the sample size and power calculations for the overall population have been published previously [17]. No tests of statistical power were applied to this analysis of the Japanese subgroup. Descriptive statistics were used to summarize the data with Kaplan–Meier (KM) estimates calculated for time-to-event variables. The Cox proportional hazard model was used to compare survival curves of OS and PFS between the 2 treatment arms.

## Results

**Fig. 1:**
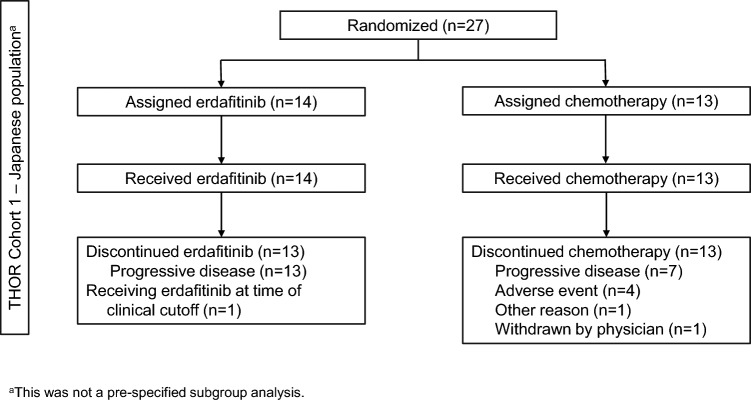
Patient disposition

Between August 6, 2018, and January 15, 2023, a total of 266 patients were randomized (erdafitinib, n = 136; chemotherapy, n = 130), of whom 27 patients (erdafitinib, n = 14; chemotherapy, n = 13) were Japanese. All Japanese patients assigned to the chemotherapy arm received docetaxel. Patient disposition is shown in Fig. 1.

### Demographic and clinical characteristics

Baseline characteristics of the Japanese patients were generally similar between the erdafitinib and chemotherapy arms, except for a greater proportion of patients with upper tract as primary site (Table 1, Supplementary Table 1). Compared with the overall population, Japanese patients had a higher proportion of males (88.9% vs 71.4%), and lower median body weight (62.0 kg vs 70.9 kg). Furthermore, Japanese patients treated with erdafitinib had a higher proportion of patients with upper tract tumors (71.4% vs 30.1%) and ECOG PS 0 (92.9 vs 46.3%) than the overall population [17].

**Table 1:** Baseline demographic and clinical characteristics^a^

Characteristics	Japanese subpopulation		Overall population	
	Erdafitinib (N = 14)	Chemotherapy (N = 13)	Erdafitinib (N = 136)	Chemotherapy (N = 130)
Age, years, median (range)	68.5 (50, 81)	70.0 (51, 84)	66.0 (32, 85)	69.0 (35, 86)
< 65 years	5 (35.7%)	2 (15.4%)	59 (43.4%)	45 (34.6%)
65–69 years	2 (14.3%)	3 (23.1%)	30 (22.1%)	23 (17.7%)
70–74 years	4 (28.6%)	5 (38.5%)	21 (15.4%)	32 (24.6%)
≥ 75 years	3 (21.4%)	3 (23.1%)	26 (19.1%)	30 (23.1%)
Sex, n (%)				
Male	12 (85.7%)	12 (92.3%)	96 (70.6%)	94 (72.3%)
Female	2 (14.3%)	1 (7.7%)	40 (29.4%)	36 (27.7%)
Weight, kg, median (range)	62.2 (42.0, 84.6)	62.0 (46.0, 72.0)	71.0 (41.0, 166.0)	70.3 (44.0, 113.0)
Primary tumor location, n (%)				
Upper tract	10 (71.4%)	6 (46.2%)	41 (30.1%)	48 (36.9%)
Lower tract	4 (28.6%)	7 (53.8%)	95 (69.9%)	82 (63.1%)
Baseline ECOG^b^, n (%)				
0	13 (92.9%)	8 (61.5%)	63 (46.3%)	51 (39.2%)
1	1 (7.1%)	5 (38.5%)	61 (44.9%)	66 (50.8%)
2	0	0	12 (8.8%)	13 (10.0%)
Visceral metastasis, n (%)	10 (71.4%)	9 (69.2%)	101 (74.3%)	97 (74.6%)
PD-(L)1 Status, n (%)				
CPS^c^ ≥ 10	0	1 (16.7%)	7 (7.3%)	11 (13.9%)
CPS^c^ < 10	9 (100.0%)	5 (83.3%)	89 (92.7%)	68 (86.1%)
FGFR alterations, n (%)				
Mutation	11 (78.6%)	10 (76.9%)	108 (79.4%)	107 (82.3%)
Fusion	3 (21.4%)	1 (7.7%)	25 (18.4%)	19 (14.6%)
Mutation and fusion	0	2 (15.4%)	2 (1.5%)	3 (2.3%)
Number of prior systemic therapy lines, n (%)				
1	4 (28.6%)	1 (7.7%)	45 (33.1%)	33 (25.4%)
2	10 (71.4%)	12 (92.3%)	90 (66.2%)	97 (74.6%)
3	0	0	1 (0.7%)	0

*CPS* combined positive score, *ECOG* eastern cooperative oncology group

^a^Percentages may not total 100 because of rounding

^b^Scores on the Eastern Cooperative Oncology Group (ECOG) scale range from 0 (no disability) to 5 (death)

^c^CPS is the number of PD-L1–staining tumor cells, lymphocytes, and macrophages, divided by the total number of viable tumor cells, multiplied by 100. Results are for patients with available data

Results of the FGFR genetic alterations analysis found that approximately 80% of Japanese patients had mutations (mostly FGFR3-Y373C and FGFR3-S249C) and a higher proportion of patients treated with erdafitinib had FGFR3 fusions (21.4% versus 7.7% in the chemotherapy arm) (Supplementary Table 2). This is similar to the pattern seen in the overall population, in which mutations (also mostly FGFR3-Y373C and FGFR3-S249C) occurred in approximately 80% of patients. Prior anti-cancer therapy consisted mainly of platinum-based chemotherapy, received by 85.2% of Japanese patients (Supplementary Table 3). Anti-PD-(L)1 therapy, most commonly pembrolizumab, was administered to all Japanese patients. Overall, the pattern of prior anti-cancer therapy use was similar between the Japanese subgroup and the overall population in Cohort 1.

As shown in Supplementary Table 4, 13 (92.9%) erdafitinib-treated patients had a serum phosphate concentration < 7.0 mg/dL at Cycle 1 Day 14 and 9 (64.3%) patients underwent up-titration. Overall, 12 (85.7%) of Japanese patients underwent at least one dose reduction, which was considerably higher than that observed in the overall population (51.1%). The extent of exposure was longer in erdafitinib-treated patients compared with chemotherapy-treated patients in the overall population and in Japanese patients in both arms compared with the overall population.

Subsequent anti-cancer therapy is shown in Supplementary Table 5. Compared with the chemotherapy arm, patients who received erdafitinib were treated with enfortumab vedotin as subsequent therapy more frequently in both the overall population and Japanese subgroup. Compared with the overall population, Japanese patients received 1–2 lines of subsequent therapy and enfortumab vedotin as subsequent therapy more frequently.

### Efficacy

**Fig. 2:**
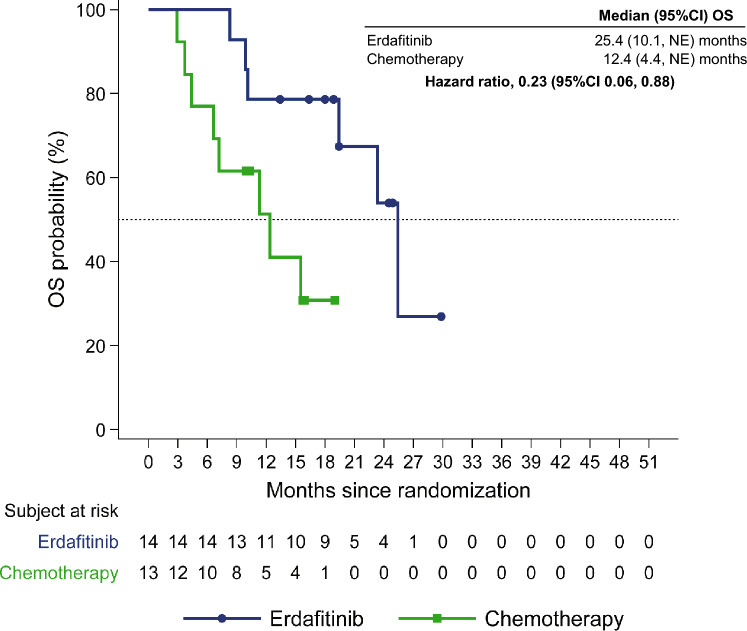
Kaplan–Meier plot of OS (primary endpoint) in Japanese patients

The median follow-up period was 19.0 months. Median OS was numerically greater in the erdafitinib arm (25.4 [95% CI 10.1, NE] months) compared with the chemotherapy arm (12.4 [4.4, NE] months) corresponding to a hazard ratio (HR) of 0.23 (0.06, 0.88; Fig. 2). OS in both arms as well as the OS difference was greater in the Japanese subgroup compared with the overall population in which the median OS in the erdafitinib arm (12.1 [95% CI 10.3, 16.4] months) and the chemotherapy arm (7.8 [6.5, 11.1] months) corresponded to a HR of 0.64 (0.47, 0.88; P = 0.005).

**Fig. 3:**
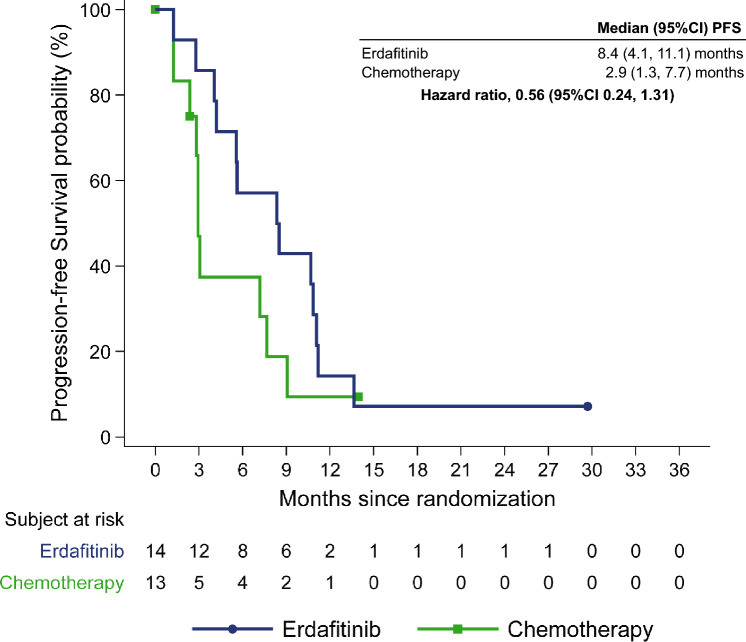
Kaplan–Meier plot of PFS (secondary endpoint) in Japanese patients

Median PFS in the erdafitinib arm (8.4 [95% CI 4.1, 11.1] months) was also greater than in the chemotherapy arm (2.9 [95% CI 1.3, 7.7] months; HR = 0.56 [95% CI 0.24, 1.31]; Fig. 3). In the overall population, the difference in PFS between the erdafitinib arm (5.6 [95% CI 4.4, 5.7] months) and chemotherapy arm (2.7 [95% CI 1.8, 3.7] months) was similar (HR = 0.58 [95% CI 0.44, 0.78]) to that observed in the Japanese subgroup.

**Fig. 4:**
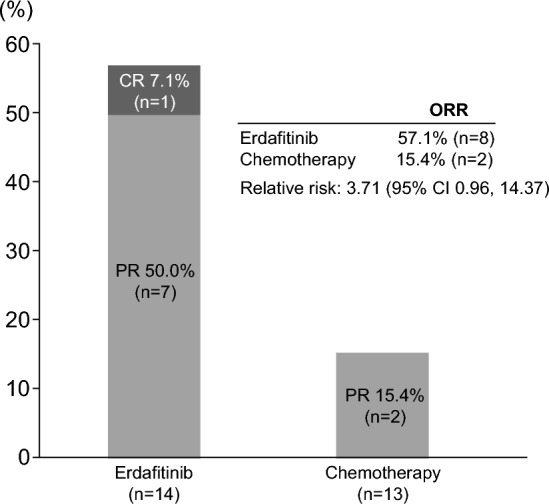
Objective response rate by investigator assessment

ORR was higher in the erdafitinib arm than in the chemotherapy arm (57.1% vs 15.4%; relative benefit 3.71; 95% CI 0.96–14.37) (Fig. 4). These results were consistent with that of the overall population, although the ORR among Japanese patients treated with erdafitinib was higher than in the overall population (45.6%). The disease control rate was higher between the Japanese subgroup treatment arms (erdafitinib, 92.9%; chemotherapy, 76.9%; relative risk: 1.21 [95% CI 0.87, 1.68]) as well as in the overall population (erdafitinib, 82.4%; chemotherapy, 43.1%; relative risk: 1.91 [95% CI 1.55, 2.35]).

### Safety

Treatment-related AEs occurred in 100% of patients in both the erdafitinib and chemotherapy arms and most were Grade 1–2 severity (Table 2). The most common treatment-related AEs in the erdafitinib arm were dysgeusia and hyperphosphatemia (71.4% each); onychomadesis (64.3%); dry mouth, diarrhea, and stomatitis (57.1% each; Table 3). The most common treatment-related AEs in the chemotherapy arm were alopecia (46.2%); neutropenia, leukopenia, and pyrexia (30.8% each); anemia, peripheral sensory neuropathy, and febrile neutropenia (23.1% each; Table 3). Grade 3–4 treatment-related AEs occurred in 6 patients (42.9%) in the erdafitinib arm and 8 patients (61.5%) in the chemotherapy arm. Treatment-related AEs leading to treatment discontinuation were not observed in the erdafitinib arm but noted in 2 patients (15.4%) in the chemotherapy arm. In contrast, treatment-related AEs leading to dose reduction or interruption were higher in the erdafitinib arm. There were no deaths related to AEs or treatment-related AEs in either treatment arm in the Japanese subgroup (Table 2). More specifically, the most common AEs leading to dose reduction (Supplementary Table 6) in the erdafitinib arm were onychomadesis (28.6%); chorioretinopathy, keratitis, and nausea (14.3% each). Similarly, the most common AEs leading to dose interruption (Supplementary Table 7) in the erdafitinib arm were onychomadesis (35.7%); paronychia (21.4%); nail discoloration, onycholysis, corneal disorder, and decreased appetite (14.3% each). AEs of clinical importance/ special interest such as nail, skin and eye toxicity including central serous retinopathy are also shown in Supplementary Table 8, where most of these toxicities were observed as Grade 1–2 severity in Japanese patients except for one case of Grade 3 skin toxicity (dry skin).

**Table 2:** Overall safety summary

Adverse events, n (%)	Japanese subpopulation		Overall population	
	Erdafitinib (N = 14)	Chemotherapy (N = 13)	Erdafitinib (N = 135)	Chemotherapy (N = 112)
Any AE	14 (100.0%)	13 (100.0%)	133 (98.5%)	109 (97.3%)
Related AEs^a^	14 (100.0%)	13 (100.0%)	131 (97.0%)	97 (86.6%)
AEs leading to death^b^	0	0	6 (4.4%)	7 (6.3%)
Related AEs^a^ leading to death^b^	0	0	1 (0.7%)	6 (5.4%)
Serious AEs	6 (42.9%)	3 (23.1%)	56 (41.5%)	47 (42.0%)
Related serious AEs	2 (14.3%)	1 (7.7%)	18 (13.3%)	27 (24.1%)
AEs leading to discontinuation of study agent	0	3 (23.1%)	19 (14.1%)	20 (17.9%)
Related AEs^a^ leading to discontinuation of study agent	0	2 (15.4%)	11 (8.1%)	15 (13.4%)
AEs leading to dose reduction of study agent	12 (85.7%)	5 (38.5%)	93 (68.9%)	27 (24.1%)
Related AEs^a^ leading to dose reduction of study agent	11 (78.6%)	4 (30.8%)	89 (65.9%)	24 (21.4%)
AEs leading to dose interruption of study agent	12 (85.7%)	7 (53.8%)	97 (71.9%)	35 (31.3%)
Related AEs^a^ leading to dose interruption of study agent	12 (85.7%)	6 (46.2%)	89 (65.9%)	22 (19.6%)
Grade 3–4 AEs	8 (57.1%)	9 (69.2%)	85 (63.0%)	72 (64.3%)
Grade 3–4 related AEs^a^	6 (42.9%)	8 (61.5%)	62 (45.9%)	52 (46.4%)
Grade 3–4 serious AEs	6 (42.9%)	2 (15.4%)	52 (38.5%)	41 (36.6%)
Grade 3–4 related serious AEs^a^	1 (7.1%)	0	16 (11.9%)	23 (20.5%)

^a^AE is categorized as related if assessed by the investigator as possibly, probably, or very likely related to study agent

^b^AEs leading to death are based on AE outcome of Fatal

**Table 3:** Treatment-related AEs with frequency ≥ 15% in either treatment arm in the Japanese subpopulation and overall population

Event	Japanese subpopulation								Overall population							
	Erdafitinib (N = 14)				Chemotherapy (N = 13)				Erdafitinib (N = 135)				Chemotherapy (N = 112)			
	Grade				Grade				Grade				Grade			
	All	1	2	≥ 3	All	1	2	≥ 3	All	1	2	≥ 3	All	1	2	≥ 3
Dysgeusia	10 (71.4%)	8 (57.1%)	1 (7.1%)	1 (7.1%)	0	0	0	0	34 (25.2%)	25 (18.5%)	8 (5.9%)	1 (0.7%)	7 (6.3%)	4 (3.6%)	3 (2.7%)	0
Hyperphosphatemia	10 (71.4%)	9 (64.3%)	0	1 (7.1%)	0	0	0	0	106 (78.5%)	70 (51.9%)	29 (21.5%)	777 (5.2%)	0	0	0	0
Onychomadesis	9 (64.3%)	3 (21.4%)	6 (42.9%)	0	2 (15.4%)	1 (7.7%)	1 (7.7%)	0	27 (20.0%)	8 (5.9%)	17 (12.6%)	2 (1.5%)	2 (1.8%)	1 (0.9%)	1 (0.9%)	0
Dry mouth	8 (57.1%)	8 (57.1%)	0	0	1 (7.7%)	1 (7.7%)	0	0	52 (38.5%)	44 (32.6%)	8 (5.9%)	0	3 (2.7%)	3 (2.7%)	0	0
Diarrhea	8 (57.1%)	5 (35.7%)	3 (21.4%)	0	3 (23.1%)	0	3 (23.1%)	0	74 (54.8%)	43 (31.9%)	27 (20.0%)	4 (3.0%)	12 (10.7%)	3 (2.7%)	6 (5.4%)	3 (2.7%)
Stomatitis	8 (57.1%)	4 (28.6%)	4 (28.6%)	0	1 (7.7%)	0	1 (7.7%)	0	62 (45.9%)	20 (14.8%)	31 (23.0%)	11 (8.1%)	13 (11.6%)	4 (3.6%)	7 (6.3%)	2 (1.8%)
Alopecia	6 (42.9%)	6 (42.9%)	0	0	6 (46.2%)	4 (30.8%)	2 (15.4%)	0	32 (23.7%)	27 (20.0%)	4 (3.0%)	1 (0.7%)	24 (21.4%)	15 (13.4%)	9 (8.0%)	0
Paronychia	6 (42.9%)	3 (21.4%)	3 (21.4%)	0	0	0	0	0	16 (11.9%)	6 (4.4%)	9 (6.7%)	1 (0.7%)	0	0	0	0
Nail discoloration	5 (35.7%)	4 (28.6%)	1 (7.1%)	0	1 (7.7%)	0	1 (7.7%)	0	24 (17.8%)	16 (11.9%)	7 (5.2%)	1 (0.7%)	2 (1.8%)	1 (0.9%)	1 (0.9%)	0
Decreased appetite	5 (35.7%)	2 (14.3%)	1 (7.1%)	2 (14.3%)	2 (15.4%)	2 (15.4%)	0	0	28 (20.7%)	15 (11.1%)	10 (7.4%)	3 (2.2%)	20 (17.9%)	8 (7.1%)	9 (8.0%)	3 (2.7%)
Dry skin	4 (28.6%)	3 (21.4%)	0	1 (7.1%)	2 (15.4%)	2 (15.4%)	0	0	30 (22.2%)	22 (16.3%)	6 (4.4%)	2 (1.5%)	4 (3.6%)	3 (2.7%)	1 (0.9%)	0
Angular cheilitis	4 (28.6%)	1 (7.1%)	3 (21.4%)	0	0	0	0	0	5 (3.7%)	2 (1.5%)	3 (2.2%)	0	0	0	0	0
Epistaxis	4 (28.6%)	4 (28.6%)	0	0	0	0	0	0	12 (8.9%)	11 (8.9%)	1 (0.7%)	0	2 (1.8%)	2 (1.8%)	0	0
Onycholysis	3 (21.4%)	0	3 (21.4%)	0	0	0	0	0	31 (23.0%)	9 (6.7%)	14 (10.4%)	8 (5.9%)	1 (0.9%)	0	1 (0.9%)	0
Nausea	3 (21.4%)	0	3 (21.4%)	0	1 (7.7%)	1 (7.7%)	0	0	14 (10.4%)	6 (4.4%)	7 (5.2%)	1 (0.7%)	22 (19.6%)	12 (10.7%)	8 (7.1%)	2 (1.8%)
Aspartate aminotransferase increased	3 (21.4%)	2 (14.3%)	1 (7.1%)	0	0	0	0	0	25 (18.5%)	18 (13.3%)	5 (3.7%)	2 (1.5%)	1 (0.9%)	1 (0.9%)	0	0
Anaemia	3 (21.4%)	1 (7.1%)	1 (7.1%)	1 (7.1%)	3 (23.1%)	0	1 (7.7%)	2 (15.4%)	16 (11.9%)	8 (5.9%)	4 (3.0%)	4 (3.0%)	31 (27.7%)	6 (5.4%)	18 (16.1%)	7 (6.3%)
Constipation	1 (7.1%)	1 (7.1%)	0	0	2 (15.4%)	2 (15.4%)	0	0	12 (8.9%)	8 (5.9%)	4 (3.0%)	0	21 (18.8%)	9 (8.0%)	10 (8.9%)	2 (1.8%)
Fatigue	1 (7.1%)	0	1 (7.1%)	0	4 (30.8%)	4 (30.8%)	0	0	18 (13.3%)	12 (8.9%)	6 (4.4%)	0	17 (15.2%)	10 (8.9%)	3 (2.7%)	2 (1.8%)
Peripheral sensory neuropathy	0	0	0	0	3 (23.1%)	2 (15.4%)	1 (7.7%)	0	4 (3.0%)	3 (2.2%)	1 (0.7%)	0	6 (5.4%)	2 (1.8%)	3 (2.7%)	1 (0.9%)
Pneumonitis	0	0	0	0	2 (15.4%)	0	2 (15.4%)	0	0	0	0	0	2 (1.8%)	0	2 (1.8%)	0
Leukopenia	0	0	0	0	4 (30.8%)	0	1 (7.7%)	3 (23.1%)	0	0	0	0	13 (11.6%)	3 (2.7%)	1 (0.9%)	9 (8.0%)
Neutropenia	0	0	0	0	4 (30.8%)	0	0	4 (30.8%)	0	0	0	0	21 (18.8%)	1 (0.9%)	5 (4.5%)	15 (13.4%)
Febrile neutropenia	0	0	0	0	3 (23.1%)	0	1 (7.7%)	2 (15.4%)	0	0	0	0	9 (8.0%)	0	1 (0.9%)	8 (7.1%)
Pyrexia	0	0	0	0	4 (30.8%)	4 (30.8%)	0	0	3 (2.2%)	1 (0.7%)	2 (1.5%)	0	7 (6.3%)	7 (6.3%)	0	0
Oedema peripheral	0	0	0	0	3 (23.1%)	1 (7.7%)	1 (7.7%)	1 (7.7%)	1 (0.7%)	1 (0.7%)	0	0	7 (6.3%)	3 (2.7%)	2 (1.8%)	2 (1.8%)
Malaise	0	0	0	0	2 (15.4%)	2 (15.4%)	0	0	0	0	0	0	3 (2.7%)	2 (1.8%)	1 (0.9%)	0
Palmar-plantar erythrodysaesthesia syndrome	2 (14.3%)	0	2 (14.3%)	0	0	0	0	0	41 (30.4%)	6 (4.4%)	22 (16.3%)	13 (9.6%)	1 (0.9%)	0	1 (0.9%)	0
Alanine aminotransferase increased	2 (14.3%)	2 (14.3%)	0	0	0	0	0	0	29 (21.5%)	18 (13.3%)	7 (5.2%)	4 (3.0%)	3 (2.7%)	2 (1.8%)	0	1 (0.9%)
Asthenia	0	0	0	0	0	0	0	0	11 (8.1%)	4 (3.0%)	6 (4.4%)	1 (0.7%)	21 (18.8%)	8 (7.1%)	11 (9.8%)	2 (1.8%)

Patients were counted only once for any given event, regardless of the number of times they actually experienced the event. The event experienced by the patient with the worst toxicity was used. If a patient had missing toxicity for a specific adverse event, the patient is only counted in the total column for that adverse event. AEs were coded using MedDRA Version 24.1

## Discussion

This subgroup analysis of the THOR Cohort 1 study confirmed that the results observed in the Japanese subgroup are consistent with those of the overall population. No notable differences were found between Japanese subgroup and overall population in the analysis of FGFR genetic alterations and prior anti-cancer therapy. Consistent efficacy of erdafitinib was observed in Japanese patients in terms of the primary endpoint of OS as well as secondary endpoints. Specifically, median OS was 25.4 months with erdafitinib compared with 12.4 months with chemotherapy. Erdafitinib also provided longer PFS (8.4 vs 2.9 months) and greater ORR (57.1% vs 15.4%) compared with chemotherapy.

The median OS and PFS, as well as ORR in Japanese patients receiving erdafitinib compared with chemotherapy were numerically greater than in the overall population. However, the small patient numbers and lack of ability to assess for statistical significance, make it difficult to conclude erdafitinib is more effective in Japanese patients. More frequent dose reductions and lower discontinuation rate due to erdafitinib-related AEs in the Japanese subgroup might also contribute to these efficacy results. In this subgroup analysis, there were notable differences in the proportion of males, patients with upper tract tumors as primary site, and better performance status (ECOG PS 0) as well as lower body weight between the Japanese subgroup and the overall population. These discrepancies may have led to the differences in survival noted between the Japanese and overall populations. In particular, a possible survival benefit in patients with upper tract tumors was noted previously in the published results for the overall population [17]. The greater proportion of patients with upper tract tumors and better performance status in the Japanese subgroup receiving erdafitinib may, therefore, have influenced better survival compared with the overall population although this should be considered cautiously given the limitations of this subgroup analysis. Better baseline performance status among Japanese patients may also have translated to improved survival compared with the overall population.

This analysis also confirmed that the safety profile of erdafitinib in Japanese patients is consistent with that observed for the overall population with no new safety signals. Treatment-related AEs leading to treatment discontinuation were not observed in the erdafitinib arm, which contrasts with the results for the overall population in which treatment discontinuation occurred in 8.1% of patients who received erdafitinib. However, treatment-related events leading to dose reduction and interruption of erdafitinib occurred in a higher proportion of Japanese patients (78.6% and 85.7%, respectively) compared with the overall population (65.9% for both outcomes) [17].

Results of this study can help clinicians further understand the potential treatment sequence of erdafitinib as therapy after anti-PD(L)1 checkpoint inhibitor in locally advanced or mUC. Other recently investigated options include enfortumab vedotin, for which the results of the phase 3 EV-301 study found that OS was longer in the enfortumab vedotin group than in the chemotherapy group in a similar setting, including among Japanese patients [9, 18]. Therefore, erdafitinib provides an alternative effective option to enfortumab vedotin but with a different safety profile to consider, most notably in relation to the potential occurrence of central serous retinopathy. In the overall population, central serous retinopathy generally resolved or was of Grade 1 severity if ongoing after the clinical cutoff date but there was no specific follow-up observation of these AEs for Japanese patients. This consideration contrasts with those of other options, such as enfortumab vedotin, in which case cutaneous reactions and peripheral neuropathy are the most frequent treatment-related AEs of special interest. Erdafitinib also has the obvious advantage of being an oral formulation that is less burdensome and more convenient for patients, involving fewer and shorter hospital visits, and which allows physicians to adjust doses more conveniently to achieve a balance between efficacy and tolerability.

Definitive data on the prevalence of FGFR alterations in Japanese patients with urothelial cancer are lacking. In a large-scale cross-sectional study, the percentage of patients in the United States with urothelial cancer and FGFR alterations was estimated to be 23.0% [19]. Another study that characterized the molecular profile of patients with urothelial cancer found FGFR3 alteration occurred in 20% of patients [20]. It is reasonable to expect that the prevalence of FGFR alterations in Japanese patients with urothelial cancer would be similar to those noted elsewhere and affect approximately one in five patients. This highlights the importance of testing for FGFR2/3 alterations, ideally at first diagnosis, to appropriately plan treatment among FGFR-positive patients. In a US healthcare claims database cohort study, uptake of erdafitinib was limited despite it being the first gene-targeted therapy for urothelial carcinoma with real-world survival outcomes similar to that of clinical trials [21]. Inadequate FGFR testing likely underlies this, and expansion of FGFRalt2/3 blood-based testing, which captures susceptible alterations at a rate similar to that of tissue testing, has the potential to improve uptake of erdafitinib in suitable patients. Such FGFR testing performed early after diagnosis of mUC should be considered to determine which patients might derive benefit from this treatment option.

This subgroup analysis is limited by various factors, including the relatively small number of patients leading to a lack of statistical power to allow for formal statistical testing. The ad hoc nature of the analysis also limits the ability of the analysis to be confirmatory.

## Conclusions

This Japanese subgroup analysis of the THOR study shows that erdafitinib improved survival and response compared to chemotherapy, with no new safety concerns. The survival benefits of erdafitinib in the Japanese subgroup supports early FGFR testing after diagnosis of mUC.

## Supplementary Information

Below is the link to the electronic supplementary material.

Supplementary file1 (DOCX 54 KB)

## Data Availability

Janssen Pharmaceutical Companies of Johnson & Johnson’s data sharing policy is available at https://www.janssen.com/clinicaltrials/transparency. As noted on this site, requests for study data access can be submitted through the Yale Open Data Access (YODA) Project site at http://yoda.yale.edu.
