# Supplementary material for: Phase 3 THOR Japanese subgroup analysis: erdafitinib in advanced or metastatic urothelial cancer and fibroblast growth factor receptor alterations
Source: Int J Clin Oncol. 2024 Jul 17;29(10):1516–27. doi: 10.1007/s10147-024-02583-3 (PMC11420312; doi:10.1007/s10147-024-02583-3)
Supplement: Supplementary file 1 — Supplementary file1 (DOCX 54 KB) [file 10147_2024_2583_MOESM1_ESM.docx]

**Title:** Phase 3 THOR Japanese subgroup analysis: Erdafitinib in advanced or metastatic urothelial cancer and fibroblast growth factor receptor alterations

**Journal name:** International Journal of Clinical Oncology

**Authors**: Nobuaki Matsubara^1^, Yuji Miura^2^, Hiroyuki Nishiyama^3^, Rikiya Taoka^4^, Takahiro Kojima^5^, Nobuaki Shimizu^6^, Jason Hwang^7^, Tatsuya Ote^8^, Ryo Oyama^9^, Kiichiro Toyoizumi^10^, Sutapa Mukhopadhyay^11^, Spyros Triantos^12^, Kris Deprince^13^, Yohann Loriot^14^

**Affiliations:**

1. Department of Medical Oncology, National Cancer Center Hospital East, 6-5-1 Kashiwanoha, Kashiwa, Chiba, 277-8577 Japan

2. Department of Medical Oncology, Toranomon Hospital, 2-2-2 Toranomon, Minato-ku, Tokyo, 105-8470 Japan

3. Department of Urology, Institute of Medicine, University of Tsukuba, 1-1-1 Tennodai, Tsukuba, Ibaraki, 305-8575 Japan

4. Department of Urology, Faculty of Medicine, Kagawa University, 1750-1 Ikenobe, Miki-cho, Kita-gun, Kagawa, 761-0793 Japan

5. Department of Urology, Aichi Cancer Center Hospital, 1-1 Kanokoden, Chikusa-ku, Nagoya, Aichi, 464-8681 Japan

6. Department of Urology, Gunma Prefectural Cancer Center, 3-39-22 Showa-machi, Maebashi, Gunma, 371-8511Japan

7. Department of Medical Affairs, Janssen Pharmaceutical K.K., 5-2-3 Nishikanda, Chiyoda-ku, Tokyo, 101-0065 Japan

8. Oncology Clinical Development Department, Clinical Science Division, Research and Development, Janssen Pharmaceutical K.K., 5-2-3 Nishikanda, Chiyoda-ku, Tokyo, 101-0065 Japan

9. Research and Development, Janssen Pharmaceutical K.K., 5-2-3 Nishikanda, Chiyoda-ku, Tokyo, 101-0065 Japan

10. Statistics and Decision Sciences, Research and Development, Janssen Pharmaceutical K. K., 5-2-3 Nishikanda, Chiyoda-ku, Tokyo, 101-0065 Japan

11. Janssen Research and Development, 920 US Highway 202 S, Raritan, New Jersey, 08807 USA

12. Janssen Research and Development, 1400 McKean Road, Spring House, PA, 19477 USA

13. Janssen Research and Development, Turnhoutseweg 30, Beerse Anterwerpen, 2340 Belgium

14. Department of Cancer Medicine, INSERM U981, Gustave Roussy, Universite Paris-Saclay, 94800 Villejuif, France

**Corresponding Author:** Nobuaki Matsubara, Department of Medical Oncology, National Cancer Center Hospital East, 6-5-1 Kashiwanoha, Kashiwa-shi, Chiba, 277-8577 Japan

**Tel:** +81-47133-1111

**E-mail:** nmatsuba@east.ncc.go.jp

# Supplementary Materials

**Table 1.** Additional baseline characteristics for Japanese subpopulation and overall population

|  | **Japanese subpopulation** | | **Overall population** | |
| --- | --- | --- | --- | --- |
| **Characteristics** | **Erdafitinib  (N=14)** | **Chemotherapy (N=13)** | **Erdafitinib  (N=136)** | **Chemotherapy (N=130)** |
| Type of histology, n (%) |  |  |  |  |
| Transitional Cell Carcinoma | 13 (92.9%) | 12 (92.3%) | 128 (94.1%) | 124 (95.4%) |
| Transitional Cell Carcinoma with minor components (<50% overall) of variant histology | 1 (7.1%) | 1 (7.7%) | 8 (5.9%) | 6 (4.6%) |
| Tumor at initial diagnosis, n (%) |  |  |  |  |
| TX | 0 | 0 | 3 (2.2%) | 10 (7.8%) |
| Ta | 0 | 0 | 0 | 1 (0.4%) |
| Ta | 1 (7.1%) | 1 (7.7%) | 13 (9.6%) | 17 (13.2%) |
| T1 | 1 (7.1%) | 2 (15.4%) | 25 (18.4%) | 26 (20.2%) |
| T2 | 2 (14.3%) | 3 (23.1%) | 45 (33.1%) | 23 (17.8%) |
| T3 | 10 (71.4%) | 6 (46.2%) | 37 (27.2%) | 32 (24.8%) |
| T4 | 0 | 1 (7.7%) | 10 (7.4%) | 17 (13.2%) |
| Presence of visceral metastases^a^ | 10 (71.4%) | 9 (69.2%) | 101 (74.3%) | 97 (74.6%) |
| Lung | 7 (50.0%) | 8 (61.5%) | 71 (52.2%) | 67 (51.5%) |
| Liver | 2 (14.3%) | 2 (15.4%) | 31 (22.8%) | 38 (29.2%) |
| Bone | 4 (28.6%) | 2 (15.4%) | 36 (26.5%) | 39 (30.0%) |
| Creatinine clearance |  |  |  |  |
| <30 mL/min | 1 (7.1%) | 1 (7.7%) | 2 (1.5%) | 1 (0.8%) |
| 30 to <60 mL/min | 9 (64.3%) | 8 (61.5%) | 57 (41.9%) | 73 (56.2%) |
| ≥60 mL/min | 4 (28.6%) | 4 (30.8%) | 77 (56.6%) | 56 (43.1%) |

^a^Number and percentages for lung, liver, and bone are based on subjects marked with “Yes” in the eCRF question “Are there currently any metastatic disease sites involving liver, lung, and/or bone?”

**Table 2.** Summary of FGFR genetic alterations

|  | **Japanese subpopulation** | | **Overall population** | |
| --- | --- | --- | --- | --- |
| **Genetic alterations** | **Erdafitinib  (N=14)** | **Chemotherapy (N=13)** | **Erdafitinib  (N=136)** | **Chemotherapy (N=130)** |
| Subjects with any FGFR alterations | 14 (100.0%) | 13 (100.0%) | 135 (99.3%) | 129 (99.2%) |
| Mutations (excluding fusions) | 11 (78.6%) | 10 (76.9%) | 108 (79.4%) | 107 (82.3%) |
| FGFR3-Y373C | 3 (21.4%) | 8 (61.5%) | 18 (13.2%) | 27 (20.8%) |
| FGFR3-S249C | 4 (28.6%) | 2 (15.4%) | 65 (47.8%) | 59 (45.4%) |
| FGFR3-G370C | 2 (14.3%) | 0 | 5 (3.7%) | 8 (6.2%) |
| FGFR3-R248C | 2 (14.3%) | 0 | 17 (12.5%) | 8 (6.2%) |
| Combinations of above | 0 | 0 | 3 (2.2%) | 5 (3.8%) |
| Fusions (excluding mutations) | 3 (21.4%) | 1 (7.7%) | 25 (18.4%) | 19 (14.6%) |
| FGFR3-TACC3 | 1 (7.1%) | 1 (7.7%) | 5 (3.7%) | 6 (4.6%) |
| FGFR3-TACC3_V1 | 1 (7.1%) | 0 | 16 (11.8%) | 10 (7.7%) |
| FGFR3-TACC3_V3 | 1 (7.1%) | 0 | 3 (2.2%) | 1 (0.8%) |
| Others | 0 | 0 | 1 (0.7%) | 2 (1.5%) |
| Mutations and fusions | 0 | 2 (15.4%) | 2 (1.5%) | 3 (2.3%) |
| FGFR3-S249C and FGFR3-TACC3_V3 | 0 | 1 (7.7%) | 2 (1.5%) | 0 |
| FGFR3-Y373C and FGFR3-TACC3_V1 | 0 | 1 (7.7%) | 0 | 1 (0.4%) |
| FGFR3-S249C and FGFR3-TACC3_V1 | 0 | 0 | 0 | 1 (0.4%) |
| FGFR3-R248C and FGFR3-TACC3_V1 | 0 | 0 | 0 | 1 (0.4%) |

**Note:** Percentages are based on the number of subjects in analysis set of the corresponding treatment group.
FGFR genetic alterations from local laboratory are used if no central laboratory data available.

**Table 3.** Prior anti-cancer therapy

|  | **Japanese subpopulation** | | **Overall population** | |
| --- | --- | --- | --- | --- |
| **Therapy** | **Erdafitinib (N=14)** | **Chemotherapy (N=13)** | **Erdafitinib (N=136)** | **Chemotherapy (N=130)** |
| Radiation therapy | 1 (7.1%) | 6 (46.2%) | 39 (28.7%) | 44 (33.8%) |
| Prior urinary surgery | 11 (78.6%) | 11 (84.6%) | 122 (89.7%) | 116 (89.2%) |
| Chemotherapy | 11 (78.6%) | 12 (92.3%) | 123 (90.4%) | 114 (87.7%) |
| Any platinum-based therapy | 11 (78.6%) | 12 (92.3%) | 122 (89.7%) | 111 (85.4%) |
| Cisplatin | 6 (42.9%) | 8 (61.5%) | 76 (55.9%) | 59 (45.4%) |
| Gem-cisplatin | 6 (42.9%) | 8 (61.5%) | 69 (50.7%) | 55 (42.3%) |
| No Gem-Cisplatin or MVAC | 0 | 0 | 0 | 0 |
| MVAC | 0 | 0 | 7 (5.1%) | 4 (3.1%) |
| Carboplatin | 3 (21.4%) | 4 (30.8%) | 37 (27.2%) | 41 (31.5%) |
| Gem-carboplatin | 3 (21.4%) | 4 (30.8%) | 36 (26.5%) | 40 (30.8%) |
| Other carboplatin | 0 | 0 | 1 (0.7%) | 3 (2.3%) |
| Multiple platinum-based therapy | 1 (7.1%) | 0 | 8 (5.9%) | 10 (7.7%) |
| No platinum-based therapy | 0 | 0 | 1 (0.7%) | 3 (2.3%) |
| Indication |  |  |  |  |
| mUC | 14 (100.0%) | 12 (92.3%) | 128 (94.1%) | 122 (93.8%) |
| Adjuvant | 3 (21.4%) | 1 (7.7%) | 17 (12.5%) | 15 (11.5%) |
| Neoadjuvant | 2 (14.3%) | 4 (30.8%) | 11 (8.1%) | 14 (10.8%) |
| Antibody drug conjugate | 0 | 0 | 0 | 2 (1.5%) |
| Enfortumab vedotin | 0 | 0 | 0 | 2 (1.5%) |
| Anti-PD-(L)1 therapy | 14 (100.0%) | 13 (100.0%) | 135 (99.3%) | 128 (98.5%) |
| Pembrolizumab | 10 (71.4%) | 12 (92.3%) | 47 (34.6%) | 47 (36.2%) |
| Avelumab | 2 (14.3%) | 0 (0.0%) | 31 (22.8%) | 28 (21.5%) |
| Not Specified | 1 (7.1%) | 1 (7.7%) | 1 (0.7%) | 2 (1.5%) |
| Durvalumab | 1 (7.1%) | 0 (0.0%) | 13 (9.6%) | 4 (3.1%) |
| Nivolumab | 0 | 0 | 11 (8.1%) | 13 (10.0%) |
| Tislelizumab | 0 | 0 | 5 (3.7%) | 3 (2.3%) |
| Cemiplimab | 0 | 0 | 0 | 1 (0.8%) |
| Sintilimab | 0 | 0 | 0 | 1 (0.8%) |
| Tocilizumab | 0 | 0 | 0 | 1 (0.8%) |

**Table 4.** Summary of study agent administration

|  | **Japanese subpopulation** | | **Overall population** | |
| --- | --- | --- | --- | --- |
|  | **Erdafitinib (N=14)** | **Chemotherapy (N=13)** | **Erdafitinib (N=135)** | **Chemotherapy (N=112)** |
| Patients with blood sample drawn for serum phosphate concentration on Day 14 of Cycle 1, n (%) | 14 (100.0%) | NA | 128 (94.8%) | NA |
| Serum phosphate level if yes |  |  |  |  |
| >9.0 mg/dL | 0 | NA | 0 | NA |
| 7.0 - 9.0 mg/dL | 1 (7.1%) | NA | 12 (8.9%) | NA |
| <7.0 mg/dL | 13 (92.9%) | NA | 116 (85.9%) | NA |
| No | 0 | NA | 7 (5.2%) | NA |
| Number of reductions in patients with up-titration | 9 (64.3%) | NA | 104 (77.0%) | NA |
| 0 | 2 (14.3%) | NA | 57 (42.2%) | NA |
| 1 | 1 (7.1%) | NA | 25 (18.5%) | NA |
| 2 | 2 (14.3%) | NA | 11 (8.1%) | NA |
| 3 | 1 (7.1%) | NA | 10 (7.4%) | NA |
| >3 | 3 (21.4%) | NA | 1 (0.7%) | NA |
| Number of reductions in patients without up-titration | 5 (35.7%) | NA | 31 (23.0%) | NA |
| 0 | 0 | NA | 9 (6.7%) | NA |
| 1 | 1 (7.1%) | NA | 8 (5.9%) | NA |
| 2 | 1 (7.1%) | NA | 8 (5.9%) | NA |
| 3 | 3 (21.4%) | NA | 6 (4.4%) | NA |
| >3 | 0 | NA | 0 | NA |
| Number of reductions in patients with dose reduction | 12 (85.7%) | NA | 69 (51.1%) | NA |
| 1 | 2 (14.3%) | NA | 33 (24.4%) | NA |
| 2 | 3 (21.4%) | NA | 19 (14.1%) | NA |
| 3 | 4 (28.6%) | NA | 16 (11.9%) | NA |
| >3 | 3 (21.4%) | NA | 1 (0.7%) | NA |
| Extent of exposure, days, median (range) | 287.5 (25, 903) | 71.0 (1, 421) | 146.0 (5, 1162) | 43.0 (1, 820) |

**Table 5.** Subsequent anti-cancer therapy

|  | **Japanese subpopulation** | | **Overall population** | |
| --- | --- | --- | --- | --- |
|  | **Erdafitinib (N=14)** | **Chemotherapy (N=13)** | **Erdafitinib (N=136)** | **Chemotherapy (N=130)** |
| Number of subjects with any subsequent therapy | 9 (64.3%) | 8 (61.5%) | 44 (32.4%) | 48 (36.9%) |
| Number of subsequent systemic therapy lines |  |  |  |  |
| 1 | 7 (50.0%) | 6 (46.2%) | 33 (24.3%) | 40 (30.8%) |
| 2 | 2 (14.3%) | 2 (15.4%) | 9 (6.6%) | 8 (6.2%) |
| 3 | 0 | 0 | 0 | 0 |
| >3 | 0 | 0 | 2 (1.5%) | 0 |
| Chemotherapy | 1 (7.1%) | 2 (15.4%) | 21 (15.4%) | 21 (16.2%) |
| Carboplatin | 1 (7.1%) | 1 (7.7%) | 8 (5.9%) | 9 (6.9%) |
| Gemcitabine | 1 (7.1%) | 1 (7.7%) | 6 (4.4%) | 10 (7.7%) |
| Paclitaxel | 0 | 1 (7.7%) | 8 (5.9%) | 6 (4.6%) |
| Other | 0 | 0 | 10 (7.4%) | 8 (6.2%) |
| Immunotherapy | 2 (14.3%) | 2 (15.4%) | 9 (6.6%) | 9 (6.9%) |
| Pembrolizumab | 2 (14.3%) | 2 (15.4%) | 4 (2.9%) | 4 (3.1%) |
| Other | 0 | 0 | 5 (3.7%) | 5 (3.8%) |
| FGFR inhibitors | 0 | 0 | 3 (2.2%) | 10 (7.7%) |
| Antibody drug conjugate | 8 (57.1%) | 5 (38.5%) | 22 (16.2%) | 14 (10.8%) |
| Enfortumab vedotin | 8 (57.1%) | 5 (38.5%) | 19 (14.0%) | 13 (10.0%) |
| Sacituzumab govitecan | 0 | 0 | 3 (2.2%) | 0 |
| Disitamab vedotin | 0 | 0 | 0 | 1 (0.8%) |
| Other systemic therapy | 0 | 0 | 2 (1.5%) | 1 (0.8%) |
| Investigational systemic therapy | 0 | 1 (7.7%) | 0 | 3 (2.3%) |
| Study Drug | 0 | 1 (7.7%) | 0 | 1 (0.8%) |
| Other | 0 | 0 | 0 | 2 (1.5%) |

**Table 6.** Adverse events leading to dose reduction

|  | **Erdafitinib (N=14)** | | | | **Chemotherapy (N=13)** | | | |
| --- | --- | --- | --- | --- | --- | --- | --- | --- |
|  | **Any grade** | **Grade 1** | **Grade 2** | **Grade ≥3** | **Any grade** | **Grade 1** | **Grade 2** | **Grade ≥3** |
| Subjects with ≥1 TEAEs leading to dose reduction | 12 (85.7%) |  |  |  | 5 (38.5%) |  |  |  |
| Chorioretinopathy | 2 (14.3%) | 2 (14.3%) | 0 | 0 | 0 | 0 | 0 | 0 |
| Keratitis | 2 (14.3%) | 2 (14.3%) | 0 | 0 | 0 | 0 | 0 | 0 |
| Corneal disorder | 1 (7.1%) | 0 | 1 (7.1%) | 0 | 0 | 0 | 0 | 0 |
| Corneal erosion | 1 (7.1%) | 1 (7.1%) | 0 | 0 | 0 | 0 | 0 | 0 |
| Macular detachment | 1 (7.1%) | 1 (7.1%) | 0 | 0 | 0 | 0 | 0 | 0 |
| Macular oedema | 1 (7.1%) | 1 (7.1%) | 0 | 0 | 0 | 0 | 0 | 0 |
| Subretinal fluid | 1 (7.1%) | 1 (7.1%) | 0 | 0 | 0 | 0 | 0 | 0 |
| Vision blurred | 1 (7.1%) | 0 | 1 (7.1%) | 0 | 0 | 0 | 0 | 0 |
| Onychomadesis | 4 (28.6%) | 1 (7.1%) | 3 (21.4%) | 0 | 0 | 0 | 0 | 0 |
| Dry skin | 1 (7.1%) | 0 | 0 | 1 (7.1%) | 0 | 0 | 0 | 0 |
| Onycholysis | 1 (7.1%) | 0 | 1 (7.1%) | 0 | 0 | 0 | 0 | 0 |
| Palmar-plantar erythrodysaesthesia syndrome | 1 (7.1%) | 1 (7.1%) | 0 | 0 | 0 | 0 | 0 | 0 |
| Skin exfoliation | 1 (7.1%) | 1 (7.1%) | 0 | 0 | 0 | 0 | 0 | 0 |
| Nausea | 2 (14.3%) | 0 | 2 (14.3%) | 0 | 0 | 0 | 0 | 0 |
| Angular cheilitis | 1 (7.1%) | 0 | 1 (7.1%) | 0 | 0 | 0 | 0 | 0 |
| Cheilitis | 1 (7.1%) | 1 (7.1%) | 0 | 0 | 0 | 0 | 0 | 0 |
| Diarrhoea | 1 (7.1%) | 1 (7.1%) | 0 | 0 | 0 | 0 | 0 | 0 |
| Dry mouth | 1 (7.1%) | 1 (7.1%) | 0 | 0 | 0 | 0 | 0 | 0 |
| Vomiting | 1 (7.1%) | 0 | 1 (7.1%) | 0 | 0 | 0 | 0 | 0 |
| Conjunctivitis | 1 (7.1%) | 0 | 1 (7.1%) | 0 | 0 | 0 | 0 | 0 |
| Nail infection | 1 (7.1%) | 0 | 1 (7.1%) | 0 | 0 | 0 | 0 | 0 |
| Paronychia | 1 (7.1%) | 1 (7.1%) | 0 | 0 | 0 | 0 | 0 | 0 |
| Dysgeusia | 1 (7.1%) | 0 | 1 (7.1%) | 0 | 0 | 0 | 0 | 0 |
| Peripheral sensory neuropathy | 0 | 0 | 0 | 0 | 1 (7.7%) | 1 (7.7%) | 0 | 0 |
| Injury corneal | 1 (7.1%) | 1 (7.1%) | 0 | 0 | 0 | 0 | 0 | 0 |
| Decreased appetite | 1 (7.1%) | 1 (7.1%) | 0 | 0 | 0 | 0 | 0 | 0 |
| Febrile neutropenia | 0 | 0 | 0 | 0 | 2 (15.4%) | 0 | 0 | 2 (15.4%) |
| Leukopenia | 0 | 0 | 0 | 0 | 2 (15.4%) | 0 | 0 | 2 (15.4%) |
| Neutropenia | 0 | 0 | 0 | 0 | 2 (15.4%) | 0 | 0 | 2 (15.4%) |
| Oedema peripheral | 0 | 0 | 0 | 0 | 1 (7.7%) | 0 | 0 | 1 (7.7%) |
| Weight increased | 0 | 0 | 0 | 0 | 1 (7.7%) | 1 (7.7%) | 0 | 0 |

**Table 7.** Adverse events leading to drug interruption

| **Adverse events** | **Erdafitinib (N=14)** | | | | **Chemotherapy (N=13)** | | | |
| --- | --- | --- | --- | --- | --- | --- | --- | --- |
|  | **Any grade** | **Grade 1** | **Grade 2** | **Grade 3** | **Any grade** | **Grade 1** | **Grade 2** | **Grade 3** |
| Subjects with ≥1 AEs leading to drug interruption | 12 (85.7%) |  |  |  | 7 (53.8%) |  |  |  |
| Onychomadesis | 5 (35.7%) | 0 | 5 (35.7%) | 0 | 0 | 0 | 0 | 0 |
| Nail discolouration | 2 (14.3%) | 1 (7.1%) | 1 (7.1%) | 0 | 0 | 0 | 0 | 0 |
| Onycholysis | 2 (14.3%) | 0 | 2 (14.3%) | 0 | 0 | 0 | 0 | 0 |
| Dry skin | 1 (7.1%) | 0 | 1 (7.1%) | 0 | 0 | 0 | 0 | 0 |
| Palmar-plantar erythrodysaesthesia syndrome | 1 (7.1%) | 0 | 1 (7.1%) | 0 | 0 | 0 | 0 | 0 |
| Rash | 1 (7.1%) | 1 (7.1%) | 0 | 0 | 0 | 0 | 0 | 0 |
| Angular cheilitis | 1 (7.1%) | 0 | 1 (7.1%) | 0 | 0 | 0 | 0 | 0 |
| Enterocolitis | 1 (7.1%) | 1 (7.1%) | 0 | 0 | 0 | 0 | 0 | 0 |
| Gastrooesophageal reflux disease | 1 (7.1%) | 0 | 1 (7.1%) | 0 | 0 | 0 | 0 | 0 |
| Mechanical ileus | 1 (7.1%) | 0 | 1 (7.1%) | 0 | 0 | 0 | 0 | 0 |
| Stomatitis | 1 (7.1%) | 0 | 1 (7.1%) | 0 | 0 | 0 | 0 | 0 |
| Tongue coated | 1 (7.1%) | 0 | 0 | 1 (7.1%) | 0 | 0 | 0 | 0 |
| Vomiting | 1 (7.1%) | 0 | 0 | 1 (7.1%) | 0 | 0 | 0 | 0 |
| Aspartate aminotransferase increased | 1 (7.1%) | 0 | 1 (7.1%) | 0 | 0 | 0 | 0 | 0 |
| Blood creatinine increased | 1 (7.1%) | 1 (7.1%) | 0 | 0 | 0 | 0 | 0 | 0 |
| C-reactive protein increased | 1 (7.1%) | 0 | 1 (7.1%) | 0 | 0 | 0 | 0 | 0 |
| Lipase increased | 1 (7.1%) | 0 | 0 | 1 (7.1%) | 0 | 0 | 0 | 0 |
| Weight decreased | 1 (7.1%) | 0 | 0 | 1 (7.1%) | 0 | 0 | 0 | 0 |
| Paronychia | 3 (21.4%) | 0 | 3 (21.4%) | 0 | 0 | 0 | 0 | 0 |
| Pneumonia | 1 (7.1%) | 1 (7.1%) | 0 | 0 | 1 (7.7%) | 1 (7.7%) | 0 | 0 |
| Urinary tract infection | 1 (7.1%) | 0 | 0 | 1 (7.1%) | 1 (7.7%) | 0 | 0 | 1 (7.7%) |
| Pyelonephritis | 1 (7.1%) | 0 | 1 (7.1%) | 0 | 0 | 0 | 0 | 0 |
| Corneal disorder | 2 (14.3%) | 0 | 2 (14.3%) | 0 | 0 | 0 | 0 | 0 |
| Chorioretinopathy | 1 (7.1%) | 1 (7.1%) | 0 | 0 | 0 | 0 | 0 | 0 |
| Keratopathy | 1 (7.1%) | 0 | 1 (7.1%) | 0 | 0 | 0 | 0 | 0 |
| Punctate keratitis | 1 (7.1%) | 0 | 1 (7.1%) | 0 | 0 | 0 | 0 | 0 |
| Subretinal fluid | 1 (7.1%) | 1 (7.1%) | 0 | 0 | 0 | 0 | 0 | 0 |
| Decreased appetite | 2 (14.3%) | 0 | 0 | 2 (14.3%) | 0 | 0 | 0 | 0 |
| Hyperphosphataemia | 1 (7.1%) | 0 | 0 | 1 (7.1%) | 0 | 0 | 0 | 0 |
| Anaemia | 1 (7.1%) | 0 | 0 | 1 (7.1%) | 2 (15.4%) | 0 | 0 | 2 (15.4%) |
| Leukocytosis | 1 (7.1%) | 0 | 1 (7.1%) | 0 | 1 (7.7%) | 0 | 1 (7.7%) | 0 |
| Pyrexia | 1 (7.1%) | 1 (7.1%) | 0 | 0 | 1 (7.7%) | 1 (7.7%) | 0 | 0 |
| Fatigue | 1 (7.1%) | 0 | 1 (7.1%) | 0 | 0 | 0 | 0 | 0 |
| Cancer pain | 1 (7.1%) | 0 | 1 (7.1%) | 0 | 0 | 0 | 0 | 0 |
| Colon cancer | 0 | 0 | 0 | 0 | 1 (7.7%) | 0 | 1 (7.7%) | 0 |
| Dysgeusia | 1 (7.1%) | 0 | 0 | 1 (7.1%) | 0 | 0 | 0 | 0 |
| Dizziness | 0 | 0 | 0 | 0 | 1 (7.7%) | 0 | 1 (7.7%) | 0 |
| Cholecystitis | 1 (7.1%) | 0 | 0 | 1 (7.1%) | 0 | 0 | 0 | 0 |
| Hypersensitivity pneumonitis | 0 | 0 | 0 | 0 | 1 (7.7%) | 0 | 1 (7.7%) | 0 |
| Pneumonitis | 0 | 0 | 0 | 0 | 1 (7.7%) | 0 | 1 (7.7%) | 0 |
| Pain in extremity | 0 | 0 | 0 | 0 | 1 (7.7%) | 1 (7.7%) | 0 | 0 |

**Table 8.** Adverse events of clinical importance / special interest

| **Event** | **Erdafitinib (N=14)** | **Chemotherapy (N=13)** |
| --- | --- | --- |
| Nail Toxicity^a^ | 13 (92.9%) | 2 (15.4%) |
| Grade ≥3 | 0 | 0 |
| Skin Toxicity^b^ | 9 (64.3%) | 3 (23.1%) |
| Grade ≥3 | 1 (7.1%) | 0 |
| Eye Toxicity (excluding central serous retinopathy)^c^ | 7 (50.0%) | 2 (15.4%) |
| Grade ≥3 | 0 | 0 |
| Central serous retinopathy^d^ | 4 (28.6%) | 0 |
| Grade ≥3 | 0 | 0 |

^a^Nail toxicity: nail bed bleeding, nail discoloration, nail disorder, nail dystrophy, nail ridging, nail toxicity, onychalgia, onychoclasis, onycholysis, paronychia, onychomadesis.

^b^Skin toxicity: blister, dry skin, erythema, hyperkeratosis, palmar, erythema, palmar-plantar erythrodysesthesla syndrome, plantar erythema, rash, rash erythematous, rash generalized, rash macular, rash maculopapular, skin atrophy, skin exfoliation, skin fissures, skin lesion, skin ulcer, toxic skin eruption, xeroderma.

^c^Eye toxicity (excluding central serous retinopathy): blepharitis, cataract, cataract subcapsular, conjunctival hemorrhage, conjunctival hyperemia, conjunctival irritation, corneal erosion, corneal infiltrates, dry eye, eye inflammation, eye irritation, eye pain, foreign body sensation in eyes, keratitis, lacrimation increased, night blindness, ocular hyperemia, photophobia, vision blurred, visual acuity reduced, visual impairment, xanthopsia, xerophthalmia, chorioretinitis, conjunctivitis, ulcerative keratitis.

^d^Central serous retinopathy: retinal detachment, vitreous detachment, retinal edema, retinopathy, chorioretinopathy, detachment of retinal pigment epithelium, detachment of macular retinal pigment epithelium, macular detachment, serous retinal detachment, subretinal fluid, retinal thickening, chorioretinitis, serous retinopathy, maculopathy, choroidal effusion.
